# A mixed methods evaluation of the Paediatric Musculoskeletal Matters (PMM) online portfolio

**DOI:** 10.1186/s12969-021-00567-5

**Published:** 2021-06-09

**Authors:** Nicola Smith, Helen E. Foster, Sharmila Jandial

**Affiliations:** 1 Translational and Clinical Research Institute, Newcastle University, Newcastle upon Tyne, UK; 2 Population and Health Sciences Institute, Newcastle University, Newcastle Upon Tyne, UK; 3 Paediatric Rheumatology, Great North Children’s Hospital, Newcastle Upon Tyne, UK

**Keywords:** Clinical education, Training, E-learning, E-resources, Telehealth, pGALS, Evaluation, Global paediatric rheumatology

## Abstract

**Background:**

The PMM Portfolio is comprised of the Paediatric Musculoskeletal Matters (PMM) website, the paediatric Gait, Arms, Legs and Spine (pGALS) app and e-learning modules (ELM). The target audiences are non-specialists in paediatric musculoskeletal medicine. Our study aimed to evaluate impact on learning and clinical practice.

**Methods:**

Mixed methods (analytics, online survey, interviews) were used with PMM and ELM registered users and purposive sampling of users using international contacts within paediatrics and paediatric rheumatology. Data was analysed using descriptive statistics and qualitative techniques. A Paired T-Test compared self-rated confidence before and after use of the PMM Portfolio.

**Results:**

There has been wide reach for all the e-resources; PMM website (662,827 hits, 262,476 users, 214 countries, data 31st July 2020); pGALS app (12,670 downloads, 70 countries, data 31st July 2020); ELM (150 users, 30 countries, data 30th May 2019). There were 164 responses (students, trainees and health care professionals) to the survey from 25 countries. Most responders deemed the PMM Portfolio useful / very useful for their learning with significantly increased self-rated confidence in their clinical examination and reasoning skills following access to the PMM website, *p* = < 0.01, pGALS app, *p* = < 0.01 and ELM, *p* = < 0.01. The most popular PMM website pages related to clinical assessment techniques (especially pGALS). There was high uptake of the pGALS app and pGALS ELM especially from trainees and allied health professionals. Many clinicians reported the PMM Portfolio to be useful when used to teach others. User feedback reported that easy navigation, open access, clinical images and cases were the most valued features. User feedback highlighted need to increase awareness of the e-resources through training programmes.

**Conclusions:**

The PMM Portfolio was developed to aid learning for clinicians who are not specialists in paediatric MSK medicine. Our evaluation demonstrates wide international reach and positive feedback on learning. The PMM Portfolio is a highly useful e-resource for paediatric rheumatologists in their teaching of others to raise awareness, facilitate early diagnosis and referral of children with suspected disease. The wide user engagement informed future PMM Portfolio development and the mixed method of evaluation is transferable to other e-resources.

**Supplementary Information:**

The online version contains supplementary material available at 10.1186/s12969-021-00567-5.

## Background

Musculoskeletal (MSK) presentations in children and young people are common with a reported prevalence of 1 in 8 [1] and a frequent cause of health care consultations increasing with age (6% of 7 year olds to 16% of 22 year olds in a cohort study from the UK) [2]. Delay in diagnosis and access to specialist care is a priority to address amongst parents [3]; unfortunately delay is often reported in conditions that present with MSK features [4–9] with adverse impact on clinical outcomes [4, 5, 10]. The reasons for delay are multifactorial [9, 11] including complex care pathways involving community and various hospital care specialties (such as general paediatrics or emergency care) [8, 11] where self-rated lack of confidence in paediatric MSK clinical skills is reported [12]. Clinicians working in the community play a crucial role to suspect MSK disease and instigate specialist referral [2, 7, 8] and family medicine practitioners are often the ‘gatekeepers’ to specialist services [13]. Unfortunately many training schemes for family medicine do not include paediatrics [14], or MSK medicine [15] despite learning needs being known [16]. Other clinicians including nurses and allied health professionals (AHP) may encounter children in their clinical practice in the community, are important in the initial care pathways and yet also have unmet needs about MSK problems [17, 18].

With these challenges in mind our group developed a portfolio of e-resources (called the PMM Portfolio), to aid learning and give support to all clinicians who play an integral role in the early recognition, diagnosis and initial care of children with MSK conditions. The target audiences of the PMM Portfolio therefore include a spectrum of clinical learners who are not ‘paediatric MSK experts’; ranging from students in medicine and nursing, trainees in family medicine and paediatrics, through to practitioners in general paediatrics, family medicine, nursing and allied health. Paediatric rheumatologists are key to the teaching of others [19–21] and especially teaching clinicians to whom children may initially present; such teaching is important to raise awareness, facilitate diagnosis and referral to specialist care. Paediatric rheumatologists are often the drivers of teaching paediatric MSK medicine at faculty and institutional level [19, 22, 23]; our evaluation aimed to support the PMM Portfolio as a valid tool to support teaching practice.

**Fig. 1:**
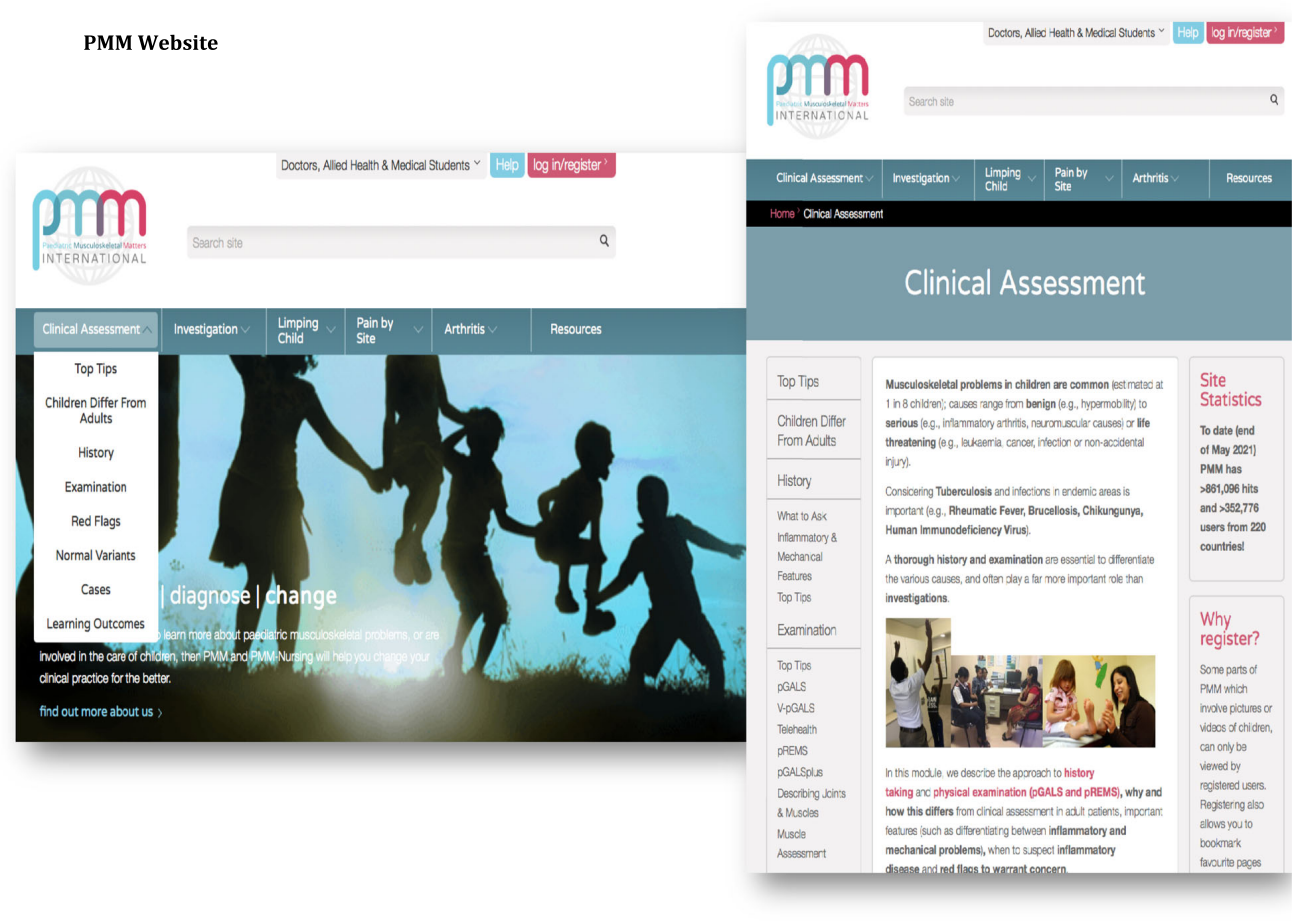
Screenshots of PMM Website

**Fig. 2:**
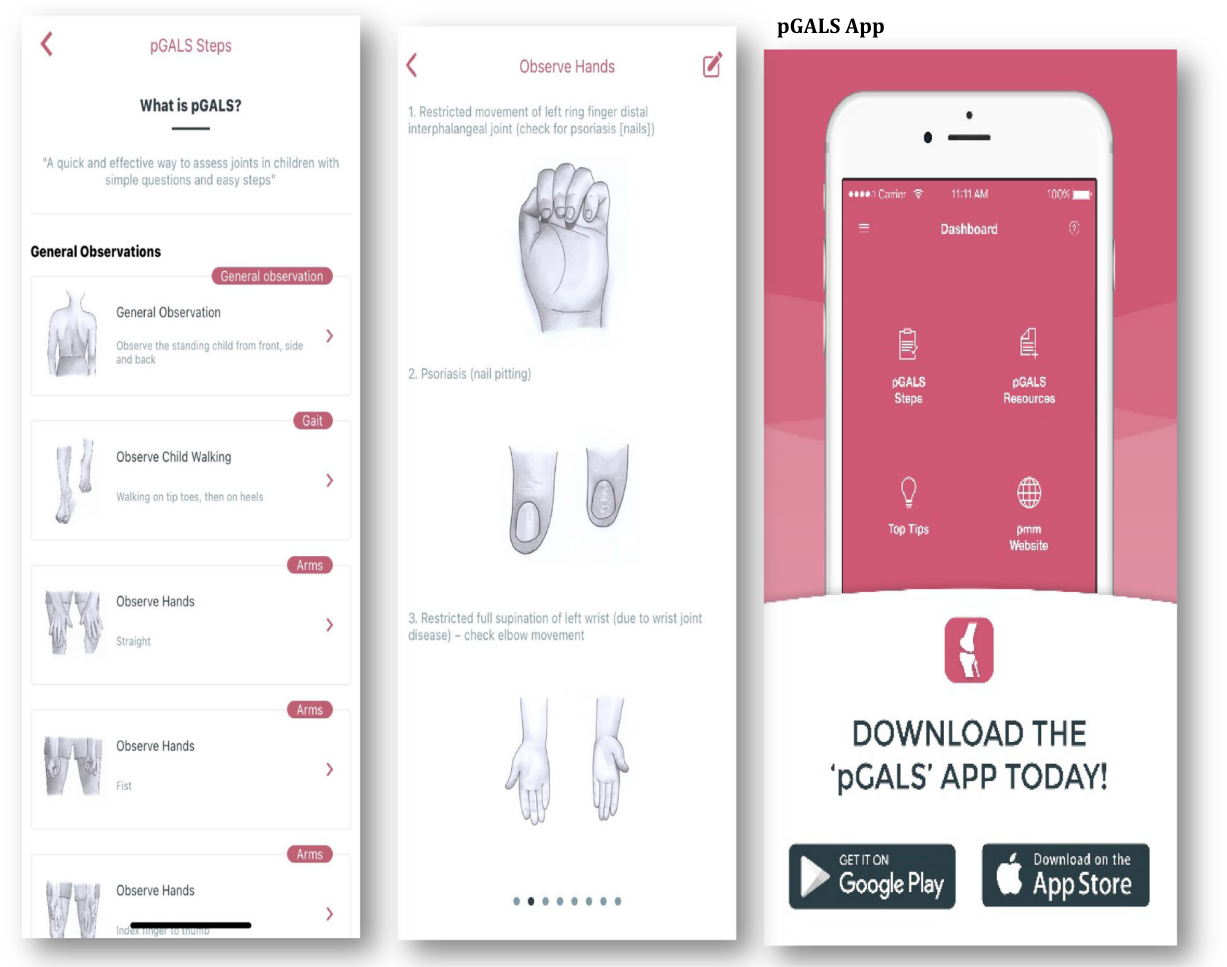
Screenshots of pGALS app

**Fig. 3:**
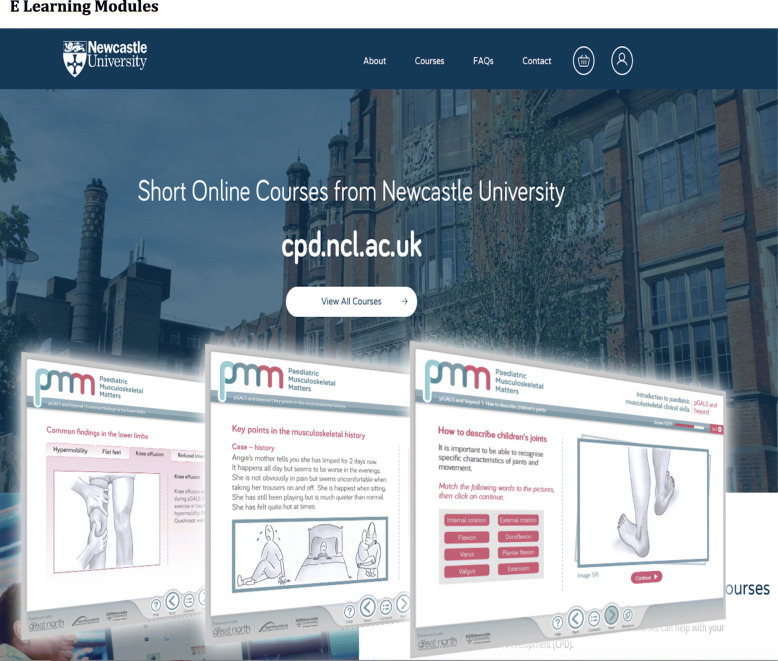
Screenshots of E Learning Modules

The PMM Portfolio encompasses the Paediatric Musculoskeletal Matters (PMM) website (launched 2014), the paediatric Gait, Arms, Legs and Spine (pGALS) app (added 2015) and e-learning modules (ELM) (added 2017) to aid signposting through the website. Figures. 1, 2 and 3 – Screenshots from the PMM Portfolio – PMM Website (Fig. 1), pGALS app (Fig. 2), ELM (Fig. 3).

The PMM website (www.pmmonline.org) is a free and open, evidence based, peer reviewed e-resource with user engagement to inform the content and website design functionality [24]. PMM Nursing (2017) is a further version of PMM to address the needs of the wider nursing community [17]. The PMM website includes an open, anonymised online survey and since 2014, 84 responses from 19 countries (data not shown but available on request), has informed PMM Portfolio development with requests for more global content and signposting. PMM International was hence developed and replaced the original PMM website in September 2018 following collaboration with paediatric rheumatologists in 11 countries (within Asia, Africa, Americas, Australasia and Europe) who developed additional content reflecting case mix and the health care in their country [25]. New content focused on infections and infection-related disease with MSK features, or differential diagnoses of rheumatic disease with further cases and images to reflect ethnic diversity. All contributions were subject to editorial review to ensure consistency of language and compliance with our governance framework [24, 25].

Paediatric Gait, Arms, Legs and Spine (pGALS) [26] is a simple MSK examination schedule useful in clinical practice [27–33] and is widely taught [34, 35]. The free pGALS app (launched in 2015) was developed with medical students at Newcastle University UK to inform format and content with exam revision notes and links to PMM website key pages [36, 37]. Language translations of pGALS produced with our PMM International collaborators (to date 20 languages) and a version for telehealth (V-pGALS) are freely available on the pGALS app and PMM website (http://www.pmmonline.org/doctor/approach-to-clinical-assessment/examination).

The PMM E-Learning Modules (ELM) aid navigation through the PMM website and were developed with our multi-professional team (including family medicine doctors, paediatricians, medical students) [35, 37]. At the time of our evaluation there were three e-modules (https://cpd.ncl.ac.uk/courses):

i) pGALS and clinical examination skills for medical students
ii) Assessment of childhood MSK presentations in family medicine
iii) The paediatricians approach to a child with fever

## Aims

This study focuses on evaluation of the PMM Portfolio to describe and understand reach and impact on learning and clinical practice. Furthermore the evaluation aimed to inform future development of the PMM Portfolio and strategy to optimise impact.

## Methods

We adopted a mixed methods approach comprising e-resource analytics, an online survey (including self-rated confidence before and after access to the e-resources) and telephone interviews to explore themes raised by the survey.

Google Analytics described access to the PMM website (*site hits, page hits, accessing countries*) and resource analytics described pGALS App Store data (*country and number of downloads*) and ELM data (*country, number of users, clinician role*).

An online survey using©Survey Monkey (see Additional file 1) explored how the PMM Portfolio e-resources impact on learning and clinical practice. The survey included sections about each of the e-resources and respondents were asked to provide feedback on all the resources (including ‘non-users’ – defined as those who had accessed some but not all the available PMM Portfolio e-resources to give insight into barriers to access). The survey was piloted and included Likert Scale questions (on perceived usefulness and impact on confidence on MSK knowledge and skills), free text options and open-ended questions to encourage comment.

Use of various recruitment sources was important to include a range of target audiences and to enable recruitment of users and non-users of the PMM Portfolio. A random selection of PMM website registered users (*n* = 450) and all the users registered for the ELM at the time of survey recruitment (*n* = 142, July 2019) were invited to complete the online survey in addition to purposive sampling amongst user groups within the UK and through our PMM global partners. Selected 1–1 telephone interviews followed the online survey to explore the findings in more detail. Interview participants comprised medical students (*n* = 2), clinical lecturer paediatricians (n = 2) and family medicine doctors involved in teaching alongside their clinical practice (n = 2); 4/6 were from the UK.

Written consent was obtained from interview participants and survey respondents consented to participation through an online response. All participant information was anonymised. Interviews were audio-recorded and transcripts anonymised before data analysis. E-resource analytic and survey data was analysed using descriptive statistics, with free-text comments and interview transcripts analysed following standard procedures for qualitative analysis, including open and focused coding, constant comparison and deviant case analysis [38]. Reflexivity was maintained throughout the analysis and writing, by recording, discussing and challenging established assumptions. A Paired T Test (©Minitab) compared self-rated confidence scores (Range: 1 not very confident – 5 very confident) before and after use of the e-resources. The study had ethical approval from Newcastle University, UK.

## Results

### Resource analytics

**Fig. 4:**
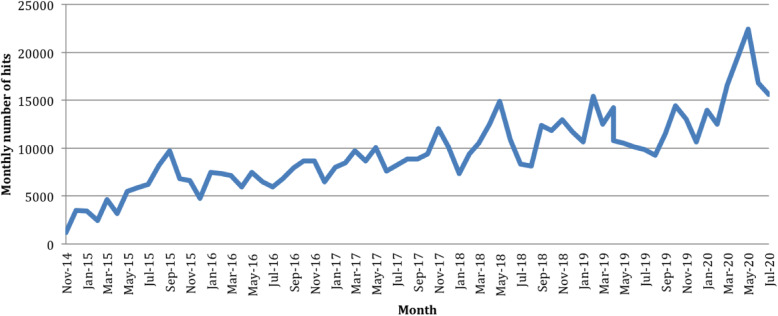
Total Number of PMM website hits each month. *Google Analytic Data from 14th November 2014 (go live date) to 31st July 2020*

The PMM website has had 662,827 hits and 262,476 users across 214 countries since launch (14th November 2014), until our data cut off for the purpose of this study (31st July 2020) - Fig. 4 and Table 1. Usage of the PMM website has grown since launch (November 2014) and furthermore following evolution to PMM International (launched September 2018). Most PMM users are from the US and UK although the total number of countries accessing PMM has markedly increased over time (see Additional file 2).

**Table 1:** Top 50 Countries Accessing the PMM Website

Country	Number of Users	Country	Number of Users
1. US	79,928	26. Spain	962
2. UK	65,417	27. Italy	890
3. Australia	15,487	28. France	865
4. India	13,895	29. Taiwan	804
5. Canada	10,796	30. Sri Lanka	793
6. Malaysia	5788	31. Colombia	788
7. Ireland	4825	32. Mexico	780
8. New Zealand	3840	33. Japan	779
9. Saudi Arabia	3534	34. China	749
10. Philippines	3050	35. Israel	749
11. South Africa	2836	36. Bangladesh	738
12. Pakistan	2343	37. Sweden	725
13. Indonesia	2187	38. Greece	608
14. Singapore	2163	39. Portugal	581
15. Thailand	1879	40. Norway	569
16. Egypt	1760	41. Poland	569
17. UAE	1491	42. Iran	542
18. Brazil	1488	43. Hungary	532
19. Netherlands	1431	44. Jordan	527
20. Hong Kong	1346	45. Belgium	513
21. Germany	1337	46. Iraq	512
22. South Korea	1284	47. Czech Republic	489
23. Nigeria	1151	48. Switzerland	467
24. Kenya	1072	49. Russia	448
25. Turkey	1001	50. Nepal	418
Total overall: 262,476 users across 214 countries			

Google Analytic Data from 14th November 2014 (go live date) to 31st July 2020.

The most popular PMM website pages (Table 2) relate to clinical assessment (*pGALS, pREMS* [39] *normal development [gait/milestones], normal variants*), frequent falls, fractures, MSK infections, limping child and guidance on when to be concerned (‘red flags’). Users spend approximately 2 min on the site (Mean: 01.58, Range: 5 s to 5 min, 53 s) and view 2 pages per session (Mean: 2.08, Range 1–24).

**Table 2:** Top 20 PMM Website Pages

Page Title	Page views
1. Gait and motor milestones	41,539
2. Frequent falls case	27,661
3. Common fractures in children	20,509
4. Septic Arthritis & Osteomyelitis	20,130
5. Limping child - abnormal gait patterns	15,327
6. pGALS	15,146
7. Tip toe walking	13,558
8. Clinical assessment - children differ from adults	12.627
9. pREMS	9261
10. Causes of foot, heel and ankle pain	8621
11. Clinical examination	8449
12. Arthritis module homepage	7965
13. Non accidental injury	7794
14. Red flags	7782
15. Personal dashboard	6394
16. Normal variants - when to refer	5902
17. Red flags - knee pain	5873
18. Kawasaki Disease	5761
19. Resources	5610
20. Clinical assessment top tips	4842

*Google Analytic Data from 14th November 2014 (go live date) to 31st July 2020.*

The pGALS app has had 12,670 downloads from 70 countries (data 31st July 2020). The iOS version had 8067 downloads from 54 countries (Top 3: China (2372), UK (1498) and US (706) (see Additional file 3). The android version had 4603 downloads from 63 countries (Top 3: UK (940), Mexico (756) and India (358)) (see Additional file 4).

The ELM data includes 150 registered users from 30 countries across Asia, Africa, America, Europe and Oceania (data 30th May 2019). Most (*n* = 101, 68%) are from the UK and Ireland with a range of clinical roles: nurse and AHP (*n* = 50), training doctors (*n* = 36) and clinicians (*n* = 35) (see Additional files 5 and 6). Most users completed one ELM (*n* = 128) and the remainder (*n* = 22) completed more than one. The ELM entitled ‘pGALS and clinical examination skills for medical students’ had the highest uptake (*n* = 130 users) followed by ‘Assessment of childhood MSK presentations in family medicine’ (*n* = 31 users), and ‘The paediatricians approach to a child with fever ‘(*n* = 11 users).

### Survey and interview data

The response rate to the survey is not clear as the link to the survey was sent to PMM collaborators and forwarded to their students and trainees. We do however know that minimum of 592 received the invite (from our random selection of PMM website users and all the ELM registered users at that time). We received 164 completed responses to the survey, hence we can assume a maximum response rate of 28%.

Survey respondents from 25 countries (across Africa, Asia, Europe, North and South America) comprised a range of roles and levels of experience within community and hospital care (see Additional files 7 and 8). Table 3 describes feedback on all three e-resources within the PMM Portfolio (*n* = 120) and an additional 44 provided feedback on 2 or less (PMM website and pGALS app (*n* = 10), PMM website and ELM (*n* = 3), PMM website alone (*n* = 31). Some had experience using the e-resources (PMM website *n* = 103/164, 63%; pGALS app *n* = 48/131, 37% ELM *n* = 50/123, 41%) and others did not have experience of one or more of the e-resources (PMM website *n* = 61/164, 37%; pGALS app *n* = 83/131, 63%; ELM *n* = 73/123, 59%). Those who had not experienced one of the e-resources were termed non-users for the purpose of our analysis but notably they all had experience of at least one of the e-resources. Users and non-users held comparable job profiles and were from a similar varied mix of countries (see Additional files 7 and 8).

**Table 3:** Use of E-Resources

Frequency of Reported Use	PMM n (%)	pGALS n (%)	Reported Use	ELM
**Use of Resource**	103 (62.80%)	48 (36.64%)	**Completed short course**	50 (40.65%)
**- Daily**	14 (13.59%)	6 (12.50%)	‑ **pGALS and clinical examination skills for medical students**	42 (34.15%)
**- Weekly**	15 (14.56%)	10 (20.83%)	‑ **Assessment of childhood MSK presentations in family medicine**	21 (17.07%)
**- Fortnightly**	7 (6.80%)	3 (6.25%)	‑ **The paediatricians approach to a child with fever**	14 (11.38%)
**- Monthly**	29 (28.16%)	11 (22.92%)		
**- Less often**	32 (31.07%)	12 (25%)		
**Other**^a^	6 (5.83%)	6 (12.50%)		
**Not Used Resource**	61 (37.20%)	83 (63.36%)	**Not completed short course**	73 (59.35%)
	Total *n* = 164	Total *n* = 131		Total *n* = 123

^a^Other included: PMM portfolio recent users accessing resources for the first time or in progress with ELM

Users of the PMM Portfolio e-resources who completed the survey comprised mainly AHP (PMM website *n* = 49/103, 48%; pGALS app *n* = 17/48, 35%; ELM *n* = 33/50, 66%), followed by general paediatricians (PMM website *n* = 13/103, 13%; pGALS app n = 7/48, 15%), paediatric rheumatologists (PMM website n = 16/103, 16%; pGALS app n = 11/48, 23%) and medical students (*n* = 4/50, 8%) for the ELM. They resided across 24 countries with highest survey uptake in India (PMM website *n* = 38/98, 39%; pGALS app n = 13/44, 30%; ELM *n* = 21/44, 48%). See Additional files 7 and 8.

Most respondents judged the e-resources to be ‘useful’ or ‘very useful’ and rated highly the fact that they could access them quickly and easily (Table 4). The main reason cited to access the PMM website and ELM were Continuing Professional Development (CPD)/Continuing Medical Education (CME), whereas supporting users to examine patients and for teaching purposes were main reasons to access the pGALS app. There was a difference by user group (see Table 5); students and trainees seeking pGALS guidance to support their clinical examination techniques and aiding revision being the main reasons to access the PMM website and pGALS app; paediatric rheumatologists cited the PMM website and pGALS app primarily for use in their teaching of others; nurses and AHP gave CPD/CME as the main reason to access the PMM website, with access to the pGALS app to support clinical examination technique and access to the ELM to aid understanding of a clinical problem.

**Table 4:** Resource Use and Impact on Education or Clinical Practice

	PMM Website	pGALS App	ELM
**How useful did you find the resource?**			
Very useful	54 (52.43%)	25 (53.19%)	23 (46.94%)
Useful	46 (44.66%)	20 (42.55%)	26 (53.06%)
Neither	2 (1.94%)	1 (2.13%)	0
Not useful	1 (0.97%)	1 (2.13%)	0
Not very useful	0	0	0
	n = 103	*n* = 47 (1 did not answer this question)	*n* = 49 (1 did not answer this question)
**Are you able to use the resource for your required purpose quickly and easily?**			
Yes	92 (89.32%)	44 (91.67%)	41 (89.13%)
No	11 (10.68%)	4 (8.33%)	5 (10.87%)
	n = 103	n = 48	*n* = 46 (4 did not answer this question)
**Do you feel the resource has or could have any impact on the clinical education of yourself or others?**			
Yes myself	69 (67.65%)	31 (65.96%)	35 (77.78%)
Yes others	26 (25.49%)	14 (29.79%)	7 (15.56%)
No	7 (6.86%)	2 (4.26%)	3 (6.67%)
	*n* = 102 (1 did not answer this question)	n = 47 (1 did not answer this question)	n = 45 (5 did not answer this question)
**Do you feel the resource has or could have any impact on your clinical practice?**			
Yes	87 (88.78%)	40 (85.11%)	40 (90.91%)
No	11 (11.22%)	7 (14.89%)	4 (9.09%)
	*n* = 98 (5 did not answer this question)	n = 47 (1 did not answer this question)	*n* = 44 (6 did not answer this question)
**Do you use any of the resources/ information available in the resource within your clinical practice?**			
Yes	66 (66%)	33 (70.21%)	33 (78.57%)
No	34 (34%)	14 (29.79%)	9 (21.43%)
	*n* = 100 (3 did not answer this question)	n = 47 (1 did not answer this question)	*n* = 42 (8 did not answer this question)

**Table 5:** Resource Use by User Group

	Training Doctors*	Clinicians	Nurses & AHP	Overall
**PMM Website Main Reason of Use**				
To find the answer to a clinical problem	7 (53.85%)	17 (45.95%)	26 (49.06%)	50 (48.54%)
To find an answer for an educational reason (e.g. essay, MCQ, exam)	5 (38.46%)	13 (35.14%)	15 (28.30%)	33 (32.04%)
For Continuing Professional Development (CPD) / For Continuing Medical Education (CME)	4 (30.77%)	15 (40.54%)	38 (71.70%)	57 (55.34%)
For academic examination preparation and revision	8 (61.54%)	8 (21.62%)	9 (16.98%)	25 (24.27%)
For teaching (of others)	5 (38.46%)	24 (64.86%)	15 (28.30%)	44 (42.71%)
To access pGALS guidance	9 (69.23%)	15 (40.54%)	15 (28.30%)	39 (37.86%)
To access pREMS guidance	6 (46.15%)	9 (24.32%)	14 (26.42%)	29 (28.16%)
Other	0	2 (5.41%) Improve skills in MSK assessment.	3 (5.66%) Improve knowledge and experience. Assess motor milestones.	5 (4.85%)
	n = 13	*n* = 37	*n* = 53	n = 103
**pGALS App Main Reason of Use**				
To help examine a patient	4 (40%)	10 (47.62%)	14 (82.35%)	28 (58.33%)
To improve my clinical examination technique	5 (50%)	10 (47.62%)	11 (64.71%)	26 (54.17%)
To improve the clinical examination technique of others	2 (20%)	12 (57.14%)	8 (47.06%)	22 (45.83%)
For academic examination preparation and revision	5 (50%)	8 (38.10%)	4 (23.53%)	17 (35.42%)
For teaching (of others)	4 (40%)	19 (90.48%)	5 (29.41%)	28 (58.33%)
	n = 10	n = 21	n = 17	n = 48
**ELM Main Reason of Use**				
To understand a clinical problem	2 (33%)	4 (40%)	25 (73.53%)	31 (62%)
To learn more for an educational reason (e.g. essay, MCQ, exam)	3 (50%)	4 (40%)	13 (38.24%)	20 (40%)
For continuing professional development (CPD)/continuing medical education (CME)	3 (50%)	5 (50%)	23 (67.65%)	31 (62%)
For academic examination preparation and revision	3 (50%)	4 (40%)	6 (17.65%)	13 (26%)
For teaching (of others)	1 (16.67)	6 (60%)	10 (29.41%)	17 (34%)
Other	0	0	1 (2.94%) Updating previous knowledge.	1 (2%)
	n = 6	n = 10	*n* = 34	n = 50

*‘Training doctor’ included medical student, general paediatric trainee, paediatric rheumatology trainee, family medicine trainee; ‘Clinician’ included, general paediatrician, paediatric rheumatologist, family medicine doctors, orthopaedic surgeon & clinical lecturer/ research fellow/medical laboratory; ‘Nurses & AHP’ included nurse/nurse practitioner, physiotherapist, podiatrist, occupational therapist, extended scope practitioner & additional needs practitioner – see Additional file 7 for breakdown

Most users reported that the PMM Portfolio had a positive impact on their current clinical practice and the learning of themselves or others (Table 4), with improved clinical skills and knowledge, aiding their teaching and increasing awareness about MSK issues amongst others cited as main benefits (Table 6).

**Table 6:** Impact of E-resources on Clinical Practice and Learning

**1. Improved clinical skills and knowledge in practice**		
PMM website	pGALS app	ELM
• Used to improve knowledge within this area and as a refresher to update and review current knowledge base. Not all countries have access to a paediatric rheumatology specialism and for these people the site enables them to view clinical cases they might otherwise not have access to within their learning environment. • Provides an intuitive source to better inform decision-making and practice, guide patient treatment and aid explaining condition to families. In particular, it informs users about systematic approach to examination and this in turn thought to enhance confidence and ability to examine children proficiently.	• Informs users about simple systematic approach to examination and serves as useful refresher or revision aid. Increased knowledge gained from the app thought to make examination easier, enhance confidence and improve examination technique. • Equips the user with the necessary knowledge and skills to discern between abnormal and normal, screen asymptomatic and symptomatic patients and distinguish musculoskeletal conditions.	• Used to expand knowledge within a particular area of interest and to consolidate and review current knowledge base or as part of CPD. • Provides content to better inform decision-making and practice and give additional reasoning that can be applied when assessing patients or explaining condition to families. Increased knowledge gained from ELM thought to aid clinical reasoning and make MSK examination easier, improve examination technique and enhance confidence particularly in relation to assessment and examination.
*“It has improved my confidence and skills to facilitate better outcomes”.* *“In my country no one have a paediatric rheumatology specialty so we can learn a lot about cases from PMM and teach our student”.* *“When I don’t have any protocol (in Brazil some hospitals doesn’t have at all) to guide me, I choose PMM to help me and solve some problems”.* *“Its easy, for free and intuitive way to find answers and guide a treatment for a patient”.* *“It will enhance my capability to check paediatrics efficiently”.*	“*Making easier the clinical examination”.* *“Increases my capability in diagnosis”.* *“Improve in terms of examination, assessment, investigation and management”.*	*“Gaining wider knowledge of signs, symptoms and examination of a child”.* *“It helped me improve my technique to perform pGALS”.* *“I think it came from wanting to consolidate what I had read. It was almost like a test to yourself. Did I actually understand what I read and what would I be inclined to do if I were presented with a certain situation”.*
**2. Improved Teaching of others**		
PMM website	pGALS app	ELM
• Used within undergraduate and trainee teaching material and students and trainees directed to site for self-directed learning or review.	• Used within undergraduate and trainee teaching material and students and trainees directed to app for self-directed learning.	• Used to prepare teaching material and inform teaching topics; and students and trainees directed to ELM for self-directed learning.
*“I refer all trainees and CME candidates to it”.* *“I can revise the knowledge of clinical history and examination skills before my teaching session”.* *“Clear, focused especially on the basics that were not taught in med school. Therefore this resource is excellent as I want to teach the topics to medical students”.*	*“I use for teaching and signpost students to it”.*	*“Acts as an introduction for me before lectures”.* *“This is very informative and attractive for us … by this we can increase our capabilities to give suggestions to others”.* *“I really want to do as much online courses as possible to have edge when enrolling for my masters”.*
**3. Raised awareness in other providers**		
PMM website	pGALS app	ELM
• Enables clinicians working in different specialties or areas to consider things from a rheumatology perspective. • Highlights key issues with MSK medicine. • Increases awareness of JIA and other rheumatological conditions in children in healthcare providers within and outside of the specialism.	• Increases knowledge in colleagues and AHP.	• Completion of the courses thought to increase awareness of rheumatological conditions in children and encourage those outside the specialism to consider MSK diagnoses when assessing patients.
*“Being an orthopaedic surgeon its useful to see problems from a rheumatological perspective”.* *“PMM is a very useful website for non paediatric rheumatologists. Highly recommend as a learning resource”.*	*“Increases knowledge in colleagues and AHP”.*	*“List out the common MSK problems of paediatrics”.*

Most users reported using the PMM Portfolio within their own learning, clinical practice or teaching of others (Table 4). The PMM website content deemed most useful related to clinical assessment and examination skills (e.g. pGALS and pREMS), normal variants, red flags, limping child guidance, links to guidelines and access to videos to demonstrate clinical skills. The pGALS guidance with illustrations and language translations were highly rated in the pGALS app. The ELM users most valued the case based presentations with discussion about when to be concerned (‘red flags’).

Self-rated confidence about MSK knowledge and skills increased for all three e-resources: rated before and after using a Likert scale range - 1 (not very confident) – 5 (very confident)*;*

- PMM Website *p* = < 0.01 t (99) = − 6.59. Before: Mean score: 3.51 (S.D. 1.19). After: Mean score: 4.23 (S.D. 0.87)
- pGALS App: *p* = < 0.01 t (46)= − 3.94. Before: Mean: 3.70 (S.D. 1.38). After: Mean: 4.30 (S.D. 1.02)
- ELM: *p* = < 0.01 (t (43)= − 4.37. Before: Mean: 3.57 (S.D. 1.21). After: Mean: 4.16 (S.D. 0.91)

Non-users of one or more of the e-resources (PMM website *n* = 61, pGALS App *n* = 83, ELM *n* = 73) cited lack of awareness of their existence being the main reason but the majority reported that following study participation, they were planning to access the e-resources for their clinical practice and / or teaching purposes.

Increasing awareness of the PMM Portfolio amongst junior doctors, nurses and AHPs was suggested to increase reach of all the e-resources. Furthermore integration of the PMM Portfolio into training or CME/CPD programmes with promotion by professional organisations were proposed to increase use of the e-resources. Expanding the range of ELM topics with apps to aid use without internet access were suggestions for future development of the PMM Portfolio.

## Discussion

The PMM Portfolio was developed to address the reality that many children will present to a myriad of clinicians and in most health care systems, not directly to a paediatric MSK specialist. Many of the clinicians involved in the early stages of the care pathway have only minimal, if any, experience or training in paediatric MSK medicine [12, 14, 15, 40]. As such, they may not easily recognise which cases are appropriate for prompt referral to a paediatric specialist. This delay in referral, which results in a delay in confirmatory diagnosis and initiation of effective treatment, can significantly impact clinical outcomes. With the aim of improving access to the “right care”, the PMM portfolio improves awareness, knowledge and clinical skills of a broad target audience, which includes medical and nursing students, medical trainees, nurses, AHPs and family medicine clinicians. The inclusion of medical and nursing students is important to instill essential skills and knowledge early in their career path [18, 34, 41].

Our evaluation demonstrates that we are reaching our target audiences and the most popular pages of the PMM website are clinical assessment, limping child, MSK infections and indicators of when to be concerned and refer to a paediatric specialist. These knowledge themes reflect essential learning in paediatric MSK medicine for medical students [42] and family medicine [16] and are key to raising awareness, facilitating early diagnosis and referral.

The mixed methods described impact with quantitative analytics (e.g. the numbers of hits, number of users, most accessed pages). Qualitative methods enabled us to explore the experiences of the users; e.g. how they use the PMM Portfolio, the impact on learning and to understand what they felt was useful and why. Our recruitment method allowed us to reach users who had used some but not all of the resources in the PMM Portfolio (e.g. those who had used the website but not the app or e-modules). Such feedback was helpful to explain why some resources had not been used and how we can further increase our reach. The methods also allowed feedback, which was used to inform iterative development of the e-resources to optimise content and format to meet the needs of the users.

Many PMM Portfolio users reported positive impact on their learning, their clinical practice and teaching of others. There was a significant increase in self-rated confidence in clinical skills and knowledge following access to all e-resources. Positive comments related to ease of use, open access and the content being at an appropriate level (either for their own learning needs or those that they teach). There was variation in access to the e-resources across user groups; e.g. students and trainees primarily using the PMM website and pGALS app for clinical skills guidance and academic examination revision, AHPs reporting use of the ELM for clinical practice and clinicians (including paediatric rheumatologists) using the PMM website and pGALS app for their teaching of others. Many users accessed the e-resources for CME/CPD.

The international uptake of the e-resources reflects the wide stakeholder engagement in the PMM Portfolio design and development [17]. Most users of the PMM website were initially from the UK and US but over time, uptake has markedly increased around the world, especially amongst trainees and AHPs. Our global partners ensure relevance of content to the target audiences and provide pGALS language translations. The global partners facilitated dissemination with the countries of several PMM global partners ranking highly in those accessing the PMM Portfolio. Dissemination has also been facilitated through endorsement by professional societies (e.g. Paediatric Rheumatology European Society (PReS), Royal College of Paediatrics and Child Health (RCPCH), Royal College of Nursing (RCN), India Paediatric Society and the National Institute for Health and Care Excellence (NICE)). The PMM Portfolio is embedded in PReS Basic Courses for paediatric rheumatology around the world, postgraduate paediatric rheumatology training programmes for paediatricians (e.g. India and Kenya), NICE Clinical Knowledge Summaries for family medicine about paediatric MSK development, RCPCH guidance for postgraduate paediatric examinations, the RCN Competency Framework for nurses and the ‘Call to Action’ strategy of the Paediatric Task Force for Global Musculoskeletal Health [43].

We firmly believe that user engagement is integral to iterative development of e-resources and optimising impact as a means of knowledge transfer [44]. User engagement provided ideas to further increase reach and these included; 1) Increasing awareness of the PMM Portfolio amongst students, trainees and AHPs; notably lack of awareness was the most cited reason amongst non-users. 2) Integration of the PMM Portfolio in training programmes through links with training bodies and increasing their exposure at CME/CPD events. 3) More ELM with topics relevant for the global context - most ELM registered users reside in UK/Ireland suggesting more work is needed to promote these internationally. 4) Offering additional e-formats to enable offline access would facilitate further their uptake. 5) Maintaining open and free access; all the e-resources are free of charge other than one ELM (‘The paediatricians approach to a child with fever’), which had higher development costs and the charge may have contributed to the lower uptake. With these suggestions in mind, work is underway to develop a PMM app to enable offline access to the PMM website content, the ELM portfolio now includes a module targeting physiotherapists (focus on gait) and a further module for school teachers is planned. To date all author contributions have been forthcoming without financial reimbursement and we gratefully acknowledge the valuable input from all our PMM partners. Funding is a major barrier to future PMM Portfolio development and we are actively working to secure sustainability and growth whilst maintaining the ethos of PMM being free and open to all.

### Limitations of our study

Our informal approach to recruitment resulted in the survey response rate being imprecise. However, this approach to recruitment enabled reach to both users and non-users of the e-resources and gave valuable insights into barriers to use and ways to encourage uptake further. There was not an even spread amongst the numbers of responders per user group or by country and this may have introduced bias. For example, there was a high proportion of survey respondents from India and amongst AHPs (who gave very favorable feedback). Their responses were nonetheless comparable to other respondent groups, so we suggest that any effect on the overall findings is minimal. Their feedback was very valuable for future work to target clinicians, especially AHPs who are integral to paediatric MSK care in areas of the world with workforce challenges [20, 40, 43, 45]. Our methods explored reach and impact on learning, clinical practice and teaching; ideally evaluation would include influence on clinical outcomes (such as access to specialist care) but given that referrals are dependent on several variables (including local referral pathways and availability of specialists), a different evaluation approach would be needed.

### Implications for research and practice

The evaluation of the PMM Portfolio is very relevant to paediatric rheumatologists who are integral to the teaching of others [19–21, 23] to raise awareness and facilitate diagnosis and referral. Paediatric rheumatologists are often the drivers and champions of paediatric MSK education at faculty and institutional level [19, 20, 23] and we hope that awareness of the PMM Portfolio and it’s positive evaluation will support its use by paediatric rheumatologists in their teaching. The PMM Portfolio is increasingly linked with educational activities delivered by paediatric rheumatologists such as the PReS Basic Courses around the world; PMM provides essential reading and preparation for the courses. Such educational activities facilitate growth of global paediatric rheumatology, especially as the content of PMM is written by paediatric rheumatologists around the world to maintain relevance to different health care contexts.

The use of e-resources in paediatric rheumatology is important; increasingly so during the COVID-19 pandemic with rapid escalation of e-learning in clinical education [46], innovative e-learning platforms [47] and the use of telemedicine [48] to reach many new users around the world. This is particularly relevant to paediatric rheumatology given the fact that many children with MSK conditions live in parts of the world (Asia and Africa) with little or no access to specialist care [49]. The PMM portfolio is therefore an exemplar model to facilitate e-learning and workforce capacity building to enable global paediatric rheumatology [43] and we anticipate a greater need to integrate e-learning with ‘face to face’ training schemes.

Our evaluation with mixed (quantitative and qualitative) methods is a valid approach applicable to other e-resources and will be useful to the paediatric rheumatology clinical educational community; understanding how to tailor e-resources to user needs and ways to optimise impact is of increasing importance given the investment in time and funding to set up and sustain e-learning programmes. Our approach highlights the importance of user engagement in iterative development, content being relevant and at the appropriate level and ways to optimise reach and impact.

## Conclusions

The PMM Portfolio is fulfilling an important educational role reaching many target user groups across the world. The PMM Portfolio continues to grow and engagement with users will facilitate future iterations maintaining relevance for the global context.

## Supplementary Information

**Additional file 1.** Blank Copy of Online Survey. Supplementary material for method.

**Additional file 2.** Top 25 Countries Accessing PMM Website by Year. Supplementary Table 1 to further illustrate results.

**Additional file 3.** pGALS App (iOS Version) Downloads by Country. Supplementary Table 2 to further illustrate results.

**Additional file 4.** pGALS App (Android Version) Downloads by Country. Supplementary Table 3 to further illustrate results.

**Additional file 5.** Job Title of Registered Users of ELM. Supplementary Table 4 to further illustrate results.

**Additional file 6.** ELM Uptake by Country. Supplementary Table 5 to further illustrate results.

**Additional file 7.** Survey Respondent Job Title or Course of Study. Supplementary Table 6 to further illustrate results.

**Additional file 8.** Countries Survey Respondent Reside In. Supplementary Table 7 to further illustrate results.

## Data Availability

To maintain anonymity no additional data is available. Participants only consented to anonymised, exemplar, extracts of the data to being shared.

## Abbreviations

AHP: Allied Health Professionals
CME: Continuing Medical Education
CPD: Continuing Professional Development
CYP: Children and Young People
ELM: E Learning Module
HCP’s: Health Care Professionals
NICE: National Institute for Health and Care Excellence
pGALS: Paediatric Gait, Arms, Legs and Spine
pREMS: Paediatric Regional Examination of the Musculoskeletal System
PReS: Paediatric Rheumatology European Society
PMM: Paediatric Musculoskeletal Matters
RCN: Royal College of Nursing
RCPCH: Royal College of Pediatrics and Child Health
