# Supplementary material for: A mixed methods evaluation of the Paediatric Musculoskeletal Matters (PMM) online portfolio
Source: Pediatr Rheumatol Online J. 2021 Jun 9;19:85. doi: 10.1186/s12969-021-00567-5 (PMC8188761; doi:10.1186/s12969-021-00567-5)
Supplement: Supplementary file 1 — Additional file 1. Blank Copy of Online Survey. Supplementary material for method. [file 12969_2021_567_MOESM1_ESM.pdf]

# E-valuate Survey

## Welcome

Thank you for agreeing to take the time to complete this survey.

We have developed three resources;

- Paediatric Musculoskeletal Matter (PMM), www.pmmonline.org (an online educational resource for all nurses and clinicians who may encounter children with musculoskeletal problems. In particular non paediatric rheumatology specialists)
- pGALS App (A musculoskeletal screening assessment app aimed at medical students & primary care)
- E Learning modules (aimed at medical students, paediatricians & primary care)

with the aim of promoting awareness and knowledge about paediatric musculoskeletal conditions to a range of healthcare professionals; and which we hope will help support our ultimate goal of supporting change in clinical practice.

These have been developed in collaboration with each resources' target audience and we now looking to obtain your feedback on their design and any impact you feel they have or could have on the clinical practice or learning of yourself or others.

This survey has four sections; in which you are asked to provide some basic information about your current role before being presented with a feedback section for each of the three resources. If you are a medical student you will also be asked an additional question at the end relating to your child health teaching. Answering the questions should take you no longer than **20 minutes** to complete.

It is hoped that your responses to this survey will highlight not only your own thoughts and any experiences you may have in relation to these resources but also help us ensure they are as useful as possible to you and any future users.

We would be grateful if you could answer all the questions honestly. There are no right or wrong answers - just your own views. Your responses will be kept anonymous and treated in confidence at all times.

## Section 1: About Me

1. Job title or course of study?

- ○ General paediatrician trainee
- ○ General paediatrician
- ○ Paediatric rheumatology trainee
- ○ Paediatric rheumatologist
- ○ Primary care trainee
- ○ Primary care doctor
- ○ Medical student
- ○ Nurse
- ○ Nursing student
- ○ Physiotherapist
- ○ Occupational therapist
- ○ Other (please specify)

2. Country of work or study?

## Section 2: Paediatric Musculoskeletal Matters (PMM)

**http://www.pmmonline.org/**

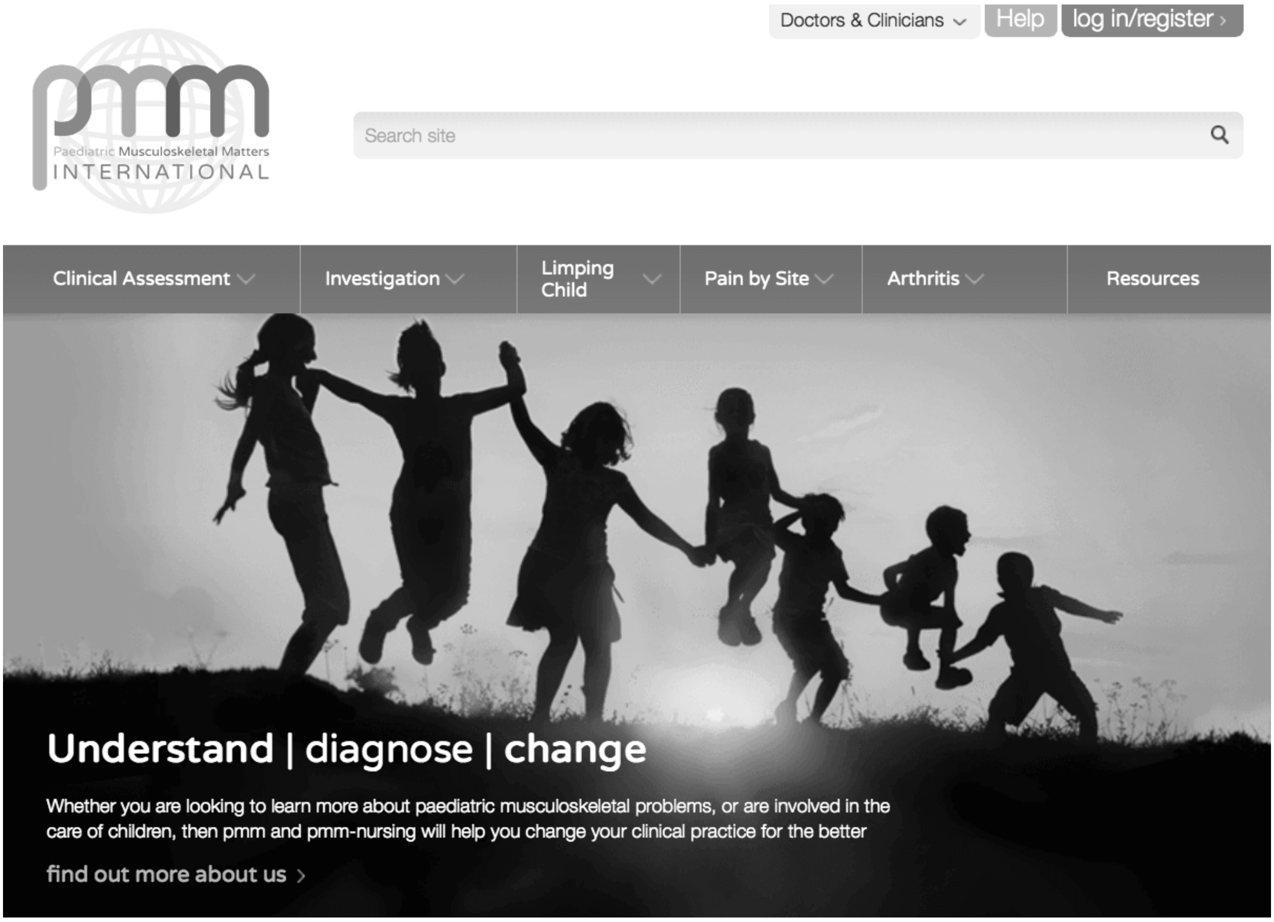


1. Have you ever accessed PMM?

- ◯ Yes
- ◯ No

## Section 2: Paediatric Musculoskeletal Matters (PMM)

2. How often do you access PMM?

- ○ Daily
- ○ Weekly
- ○ Fortnightly
- ○ Monthly
- ○ Less often
- ○ Other (please specify)

3. What is your main reason(s) for using PMM? [Please select all that apply]

- ☐ To find the answer to a clinical problem
- ☐ To find an answer for an educational reason (e.g. essay, MCQ, exam)
- ☐ For Continuing Professional Development (CPD) / Continuing Medical Education (CME)
- ☐ For exam revision
- ☐ For teaching
- ☐ To access pGALS guidance
- ☐ To access pREMS guidance
- ☐ Other (please specify)

4. How useful do you find PMM?

- ○ Not very useful
- ○ Not useful
- ○ Neither
- ○ Useful
- ○ Very useful

Please explain

5. What content within PMM is most useful for you personally? [Please select all that apply] – this list is based on the most accessed pages from PMM website

- ☐ Clinical assessment – history taking
- ☐ Clinical examination skills e.g. pGALS, pREMS
- ☐ Link to pGALS app
- ☐ Normal development and milestones
- ☐ How children differ from adults (arthritis presentations)
- ☐ Limping child differential stratified by age
- ☐ Red flags symptoms and signs
- ☐ Septic arthritis and osteomyelitis – key features
- ☐ Investigations (e.g. autoantibodies, reference ranges)
- ☐ Frequent falls differential
- ☐ Common fractures in children
- ☐ Causes of foot, heel and ankle pain
- ☐ Causes of hip pain
- ☐ Causes of knee pain
- ☐ Causes of back pain
- ☐ Joint contractures – differential and approach to assessment
- ☐ Arthritis – differential and list of causes
- ☐ JIA subtypes
- ☐ Non accidental injury – red flags
- ☐ Links to NICE guidance
- ☐ Top Tips
- ☐ Link to e modules and other resources
- ☐ Other (please specify)

6. Is there any additional content you feel should be included?

- ○ Yes
- ○ No

If yes please provide detail

7. Are you able to use PMM for your required purpose quickly and easily?

- ◯ Yes
- ◯ No

Please explain

8. Please list any potential barriers to using PMM

9. Do you feel PMM has or could have any impact on the medical education of yourself or others?

- ◯ Yes myself
- ◯ Yes others
- ◯ No

Please explain

10. Do you feel PMM has or could have any impact on your clinical practice?

- ◯ Yes
- ◯ No

Please explain

11. Do you use any of the resources available at PMM within your clinical practice?

- ◯ Yes
- ◯ No

If yes, which sections do you use?

12. How would you rate your confidence in diagnosing paediatric musculoskeletal problems before using PMM on a scale of 1 (not very confident) to 5 (very confident)?

- ◯ 1 - Not very confident
- ◯ 2
- ◯ 3
- ◯ 4
- ◯ 5 - Very confident

13. How would you rate your confidence in diagnosing paediatric musculoskeletal problems after using PMM on a scale of 1 (not very confident) to 5 (very confident)?

- ◯ 1 - Not very confident
- ◯ 2
- ◯ 3
- ◯ 4
- ◯ 5 - Very confident

14. What could we do to increase the impact of PMM?

15. What do you think are the barriers to changing practice with PMM?

16. What could we do to reduce these barriers?

17. How can we increase the use of PMM?

18. Is there anything we can do to improve your experience of PMM?

19. Any final comments

## Section 2: Paediatric Musculoskeletal Matters (PMM)

2. Why have you not used PMM?

- ○ Unaware of its existence
- ○ Experienced problems accessing the site
- ○ Not useful for me
- ○ Other (please specify)

3. Do you plan to use PMM in the future?

- ○ Yes
- ○ No

## Section 2: Paediatric Musculoskeletal Matters (PMM)

4. What do you think will be your main reason(s) for using PMM? [Please select all that apply]

- [ ] To find the answer to a clinical problem
- [ ] To find an answer for an educational reason (e.g. essay, MCQ, exam)
- [ ] For Continuing Professional Development (CPD) / Continuing Medical Education (CME)
- [ ] For exam revision
- [ ] For teaching
- [ ] To access pGALS guidance
- [ ] To access pREMS guidance
- [ ] Other (please specify)

5. What content within PMM do you think would be most useful for you personally? [Please select all that apply]

- [ ] Clinical assessment – history taking
- [ ] Clinical examination skills e.g. pGALS, pREMS
- [ ] Link to pGALS app
- [ ] Normal development and milestones
- [ ] How children differ from adults (arthritis presentations)
- [ ] Limping child differential stratified by age
- [ ] Red flags symptoms and signs
- [ ] Septic arthritis and osteomyelitis – key features
- [ ] Investigations (e.g. autoantibodies, reference ranges)
- [ ] Frequent falls differential
- [ ] Common fractures in children
- [ ] Causes of foot, heel and ankle pain
- [ ] Causes of hip pain
- [ ] Causes of knee pain
- [ ] Causes of back pain
- [ ] Joint contractures – differential and approach to assessment
- [ ] Arthritis – differential and list of causes
- [ ] JIA subtypes
- [ ] Non accidental injury – red flags
- [ ] Links to NICE guidance
- [ ] Top Tips
- [ ] Link to e modules and other resources
- [ ] Other (please specify)

6. Please list any potential barriers to using PMM

7. Do you feel PMM could have any impact on the medical education of yourself or others?

- ○ Yes myself
- ○ Yes others
- ○ No

Please explain

8. Do you feel PMM could have any impact on your clinical practice?

- ○ Yes
- ○ No

Please explain

9. What could we do to increase the impact of PMM?

10. What do you think are the barriers to changing practice with PMM?

11. What could we do to reduce these barriers?

12. How can we increase the use of PMM?

13. Is there anything we can do to improve your experience of PMM?

14. Any final comments

## Section 2: Paediatric Musculoskeletal Matters (PMM)

4. What (if anything) could we do to encourage you to use PMM in the future?

5. Any final comments?

## Section 3: pGALS App

**Available to download on Apple App Store and Google Play**

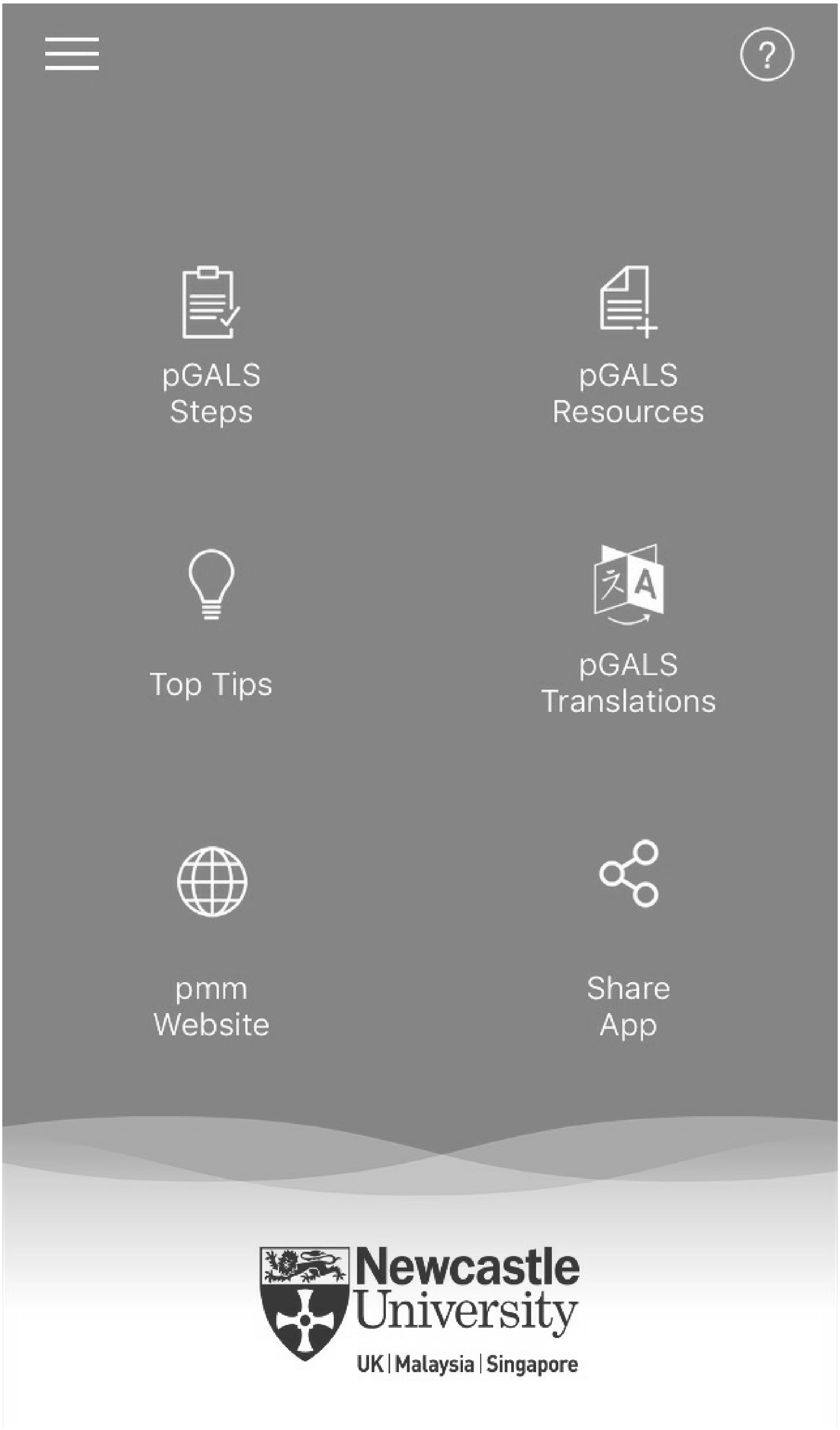
pGALS Steps
pGALS Resources
Top Tips
pGALS Translations
pmm Website
Share App
Newcastle University
UK | Malaysia | Singapore

1. Have you ever used the pGALS App?

- ◯ Yes
- ◯ No

## Section 3: pGALS App

2. How often do you use the pGALS App?

- ◯ Daily
- ◯ Weekly
- ◯ Fortnightly
- ◯ Monthly
- ◯ Less often
- ◯ Other (please specify)

3. What is your main reason(s) for using this app? ? [Please select all that apply]

- ☐ To help examine a patient
- ☐ To improve my examination technique
- ☐ To improve the examination technique of others
- ☐ For exam revision
- ☐ For teaching
- ☐ Other (please specify)

4. How useful do you find the pGALS app?

- ◯ Not very useful
- ◯ Not useful
- ◯ Neither
- ◯ Useful
- ◯ Very useful

Please explain

5. What content within the app is most useful for you personally?[Please select all that apply]

- [ ] pGALS steps
- [ ] pGALS resources
- [ ] pGALS paper proforma
- [ ] Revision checklist
- [ ] pGALS recording
- [ ] pGALS translations
- [ ] Top tips: Practical tips
- [ ] Top tips: 10 top tips for clinical assessment
- [ ] Top tips: 10 top tips for clinical examination
- [ ] Top tips: 10 top tips for history taking
- [ ] Top tips: 10 top tips for hypermobility
- [ ] Top tips: 10 top tips for limping child
- [ ] Link to PMM website
- [ ] Timed test mode

6. Is there any additional content you feel should be included?

- ○ Yes
- ○ No

If yes please provide detail

7. Are you able to use the pGALS app for your required purpose quickly and easily?

- ○ Yes
- ○ No

Please explain

8. Please list any potential barriers to using the pGALS app

9. Do you feel the pGALS app has or could have any impact on the medical education of yourself or others?

- ◯ Yes myself
- ◯ Yes others
- ◯ No

Please explain

10 Do you feel the pGALS app has or could have any impact on your clinical practice?

- ◯ Yes
- ◯ No

Please explain

11. Do you use any of the resources available in the pGALS app within your clinical practice?

- ◯ Yes
- ◯ No

If yes which do you use?

12. How would you rate your confidence in musculoskeletal examination <u>before</u> using the pGALS app on scale of 1 (not very confident) to 5 (very confident)?

- ◯ 1 - Not very confident
- ◯ 2
- ◯ 3
- ◯ 4
- ◯ 5 - Very confident

13. How would you rate your confidence in musculoskeletal examination after using the pGALS app on scale of 1 (not very confident) to 5 (very confident)?

- ◯ 1 - Not very confident
- ◯ 2
- ◯ 3
- ◯ 4
- ◯ 5 - Very confident

14. What could we do to increase the impact of the pGALS app?

15. What do you think are the barriers to changing practice with the pGALS app?

16. What could we do to reduce these barriers?

17. How can we increase the use of the pGALS app?

18. Is there anything we can do to improve your experience of the pGALS app?

19. Any final comments

## Section 3: pGALS App

2. Why have you not used the pGALS app?

- [ ] Unaware of its existence
- [ ] Experienced problems downloading the app
- [ ] Experienced problems using the app
- [ ] Device is not compatible with Google Play or Apple Store
- [ ] Not useful for me
- [ ] Other (please specify)

3. Do you plan to use the pGALS app in the future?

- ( ) Yes
- ( ) No

## Section 3: pGALS App

4. What do you think will be your main reason(s) for using the pGALS app? [Please select all that apply]

- ☐ To help examine a patient
- ☐ To improve my examination technique
- ☐ To improve the examination technique of others
- ☐ For exam revision
- ☐ For teaching
- ☐ Other (please specify)

5. What content within the pGALS app do you think would be most useful for you personally? [Please select all that apply]

- ☐ pGALS steps
- ☐ pGALS resources
- ☐ pGALS paper proforma
- ☐ revision checklist
- ☐ pGALS recording
- ☐ pGALS translations
- ☐ Top tips: Practical tips
- ☐ Top tips: 10 top tips for clinical assessment
- ☐ Top tips: 10 top tips for clinical examination
- ☐ Top tips: 10 top tips for history taking
- ☐ Top tips: 10 top tips for hypermobility
- ☐ Top tips: 10 top tips for limping child
- ☐ Link to PMM website
- ☐ Timed test mode
- ☐ Other (please specify)

6. Please list any potential barriers to using the pGALS app.

7. Do you feel the pGALS app could have any impact on the medical education of yourself or others?

- ◯ Yes myself
- ◯ Yes others
- ◯ No

Please explain

[ ]

8. Do you feel the pGALS app could have any impact on your clinical practice?

- ◯ Yes
- ◯ No

Please explain

[ ]

9. What could we do to increase the impact of the pGALS app?

[ ]

10. What do you think are the barriers to changing practice with the pGALS app?

[ ]

11. What could we do to reduce these barriers?

[ ]

12. How can we increase the use of the pGALS app?

[ ]

13. Is there anything we can do to improve your experience of the pGALS app?

[ ]

14. Any final comments

## Section 3: pGALS App

4. What (if anything) could we do to encourage you to use the pGALS app in the future?

5. Any final comments?

## Section 4: E Learning Modules

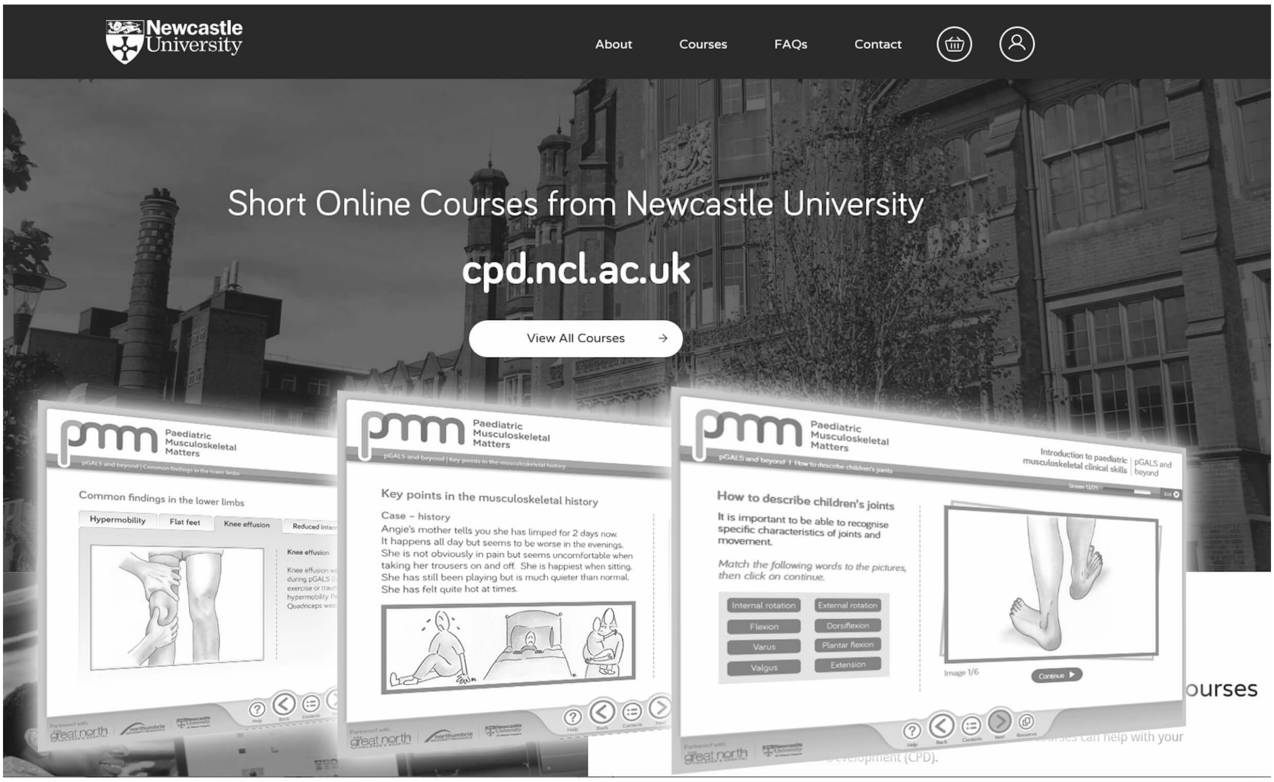


1. Have you completed any of the short online courses offered by Newcastle University?

- [ ] Yes - Introduction to Paediatric Musculoskeletal Clinical Skills – pGALS
- [ ] Yes - Paediatric Musculoskeletal Medicine in Primary Care - A Guide for GPs
- [ ] Yes - The Child with Fever - Infection or not?
- [ ] No

## Section 4: E Learning Modules

2. How did you find out about the course

- ☐ Word of mouth
- ☐ Recommendation from a colleague/fellow student
- ☐ Advertising from Newcastle university
- ☐ Advertising from other source (please specify)
- ☐ Other (please specify)

3. What was your main reason(s) for completing the course(s)? ? [Please select all that apply]

- ☐ To understand a clinical problem
- ☐ To learn more for an educational reason (e.g. essay, MCQ, exam)
- ☐ For Continuing Professional Development (CPD) / Continuing Medical Education (CME)
- ☐ For exam revision
- ☐ For teaching
- ☐ Other (please specify)

4. How useful did you find the course(s)?

- ○ Not very useful
- ○ Not useful
- ○ Neither
- ○ Useful
- ○ Very useful

Please explain

5. What content within the course(s) was most useful for you personally?

6. Is there any additional content you feel should be included?

- ◯ Yes
- ◯ No

If yes please provide detail

7. Were you able to use the course(s) for your required purpose quickly and easily?

- ◯ Yes
- ◯ No

Please explain

8. Did you pay for the course(s)?

- ◯ Yes
- ◯ No

If so how much?

9. Would you pay for any courses like this in the future?

- ◯ Yes
- ◯ No

If so how much?

10. Please list any potential barriers to completing the course(s)

11. Do you feel the course(s) has or could have any impact on the medical education of yourself or others?

- ◯ Yes myself
- ◯ Yes others
- ◯ No

Please explain

12. Do you feel the course(s) has or could have any impact on your clinical practice?

- ◯ Yes
- ◯ No

Please explain

13. Do you use any of the information from the course(s) within your clinical practice?

- ◯ Yes
- ◯ No

If yes which information do you use?

14. How would you rate your confidence in paediatric musculoskeletal medicine <u>before</u> completing the course(s) on scale of 1 (not very confident) to 5 (very confident)?

- ◯ 1 - Not very confident
- ◯ 2
- ◯ 3
- ◯ 4
- ◯ 5 - Very confident

15. How would you rate your confidence in paediatric musculoskeletal medicine <u>after</u> completing the course(s) on scale of 1 (not very confident) to 5 (very confident)?

- ◯ 1 - Not very confident
- ◯ 2
- ◯ 3
- ◯ 4
- ◯ 5 - Very confident

16. What could we do to increase the impact of the course(s)?

17. What do you think are the barriers to changing practice with the course(s)?

18. What could we do to reduce these barriers?

19. How can we increase the use of the course(s)?

20. Is there anything we can do to improve your experience of the course(s)?

21. Are there any additional courses you think we should develop in relation to the area of paediatric musculoskeletal medicine?

22. Any final comments

## Section 4: E Learning Modules

2. Why have you not completed the course(s)?

- ◯ Unaware of existence
- ◯ Experienced problems registering for the course(s)
- ◯ Experienced problems using the course(s)
- ◯ Not useful for me
- ◯ Other (please specify)

3. Do you plan to complete any of these courses in the future?

- ◯ Yes
- ◯ No

Please explain

## Section 4: E Learning Modules

4. Which course(s) would you be interested in completing?

- [ ] Introduction to Paediatric Musculoskeletal Clinical Skills – pGALS
- [ ] Paediatric Musculoskeletal Medicine in Primary Care - A Guide for GPs
- [ ] The Child with Fever - Infection or not?

5. What do you think will be your main reason(s) for completing the course(s)? [Please select all that apply]

- [ ] To understand a clinical problem
- [ ] To learn more for an educational reason (e.g. essay, MCQ, exam)
- [ ] For Continuing Professional Development (CPD) / Continuing Medical Education (CME)
- [ ] For exam revision
- [ ] For teaching
- [ ] Other (please specify)

6. What content within the course(s) do you think would be most useful for you personally?

7. Would you pay for any courses like this in the future?

- ○ Yes
- ○ No

If so how much

8. Please list any potential barriers to completing the course(s).

9. Do you feel the course(s) could have any impact on the medical education of yourself or others?

- ◯ Yes myself
- ◯ Yes others
- ◯ No

Please explain

10. Do you feel the course(s) could have any impact on your clinical practice?

- ◯ Yes
- ◯ No

Please explain

11. What could we do to increase the impact of the course(s)?

12. What do you think are the barriers to changing practice with the course(s)?

13. What could we do to reduce these barriers?

14. How can we increase the use of the course(s)?

15. Is there anything we can do to improve your experience of the course(s)?

16. Are there any additional courses you think we should develop in relation to the area of paediatric musculoskeletal medicine?

17. Any final comments

## Section 4: E Learning Modules

4. What (if anything) could we do to encourage you to complete the course(s) in the future?

5. Any final comments?

## Medical Student Specific Questions

Finally, are you a medical student?

- ◯ Yes
- ◯ No

## Medical Student Specific Questions

Learning outcomes for medical students in relation to paediatric musculoskeletal medicine include:

- Knowing about and being able to perform pGALS as a core skill
- Knowing about limping child as a core presentation

### Have you had any teaching of pGALS or limping child in your child health teaching?

- ◯ Yes
- ◯ No

**If yes:**

### What format did this take?

- ☐ Lecture
- ☐ Seminar
- ☐ Workshop
- ☐ Bedside teaching
- ☐ Self directed learning with resources provided
- ☐ Self directed learning with no resources provided
- ☐ Other (please specify)

### Were of the resources discussed today useful or would they be useful to support this learning?

| | Was useful | Would be useful | Was not or would not be useful |
|---|---|---|---|
| PMM | ◯ | ◯ | ◯ |
| pGALS App | ◯ | ◯ | ◯ |
| E Learning Module: Introduction to Paediatric Musculoskeletal Clinical Skills – pGALS | ◯ | ◯ | ◯ |
| E Learning Module:Paediatric Musculoskeletal Medicine in Primary Care - A Guide for GPs | ◯ | ◯ | ◯ |
| E Learning Module:The Child with Fever - Infection or not | ◯ | ◯ | ◯ |

Please describe how and why.

E-valuate Survey

## Thank you

You have now come to the end of the survey. Thank you once again for taking the time to complete these questions.

At this stage in the project we have used surveys to explore the views of our resource's target audiences. This in addition to the data we will collect during the next stages of the project will be used to help ensure the resources are as beneficial to their users as possible. This research is currently in the early stages but if you would like to be informed once the results are published please provide the project's research associate Dr Nicola Smith with your contact details either via email (nicola.smith@newcastle.ac.uk) or below.

Likewise, if you are interested in taking part in or would like more information about the next stage(s) of the project please provide your contact details in the box below or contact Nicola directly.

**I would like to be contacted when the results are available.**

- ○ Yes
- ○ No

**I am interested in taking part in or would like more information about the next stage(s) of the project.**

- ○ Yes
- ○ No

**Contact Details**

**Name** ______________________

**Email Address** ______________________

All of the information gathered in this study will remain confidential and your identity will not be referred to in any written report following this study. All data will be stored in line with the Data Protection Act and will be compliant with General Data Protection Regulation (GDPR) guidance.

If you decide you would no longer like to be part of the study, you can withdraw from the study at any time by contacting a member of the research team. The research team will keep the data already collected up to that point in an anonymised form. Likewise if you have any questions or require any further information please feel free to contact Dr Nicola Smith.

Thank you for your participation.
