# Supplementary material for: A mixed methods evaluation of the Paediatric Musculoskeletal Matters (PMM) online portfolio
Source: Pediatr Rheumatol Online J. 2021 Jun 9;19:85. doi: 10.1186/s12969-021-00567-5 (PMC8188761; doi:10.1186/s12969-021-00567-5)
Supplement: Supplementary file 2 — Additional file 2. Top 25 Countries Accessing PMM Website by Year. Supplementary Table 1 to further illustrate results. [file 12969_2021_567_MOESM2_ESM.docx]

**Additional Table 1: Top 25 Countries Accessing PMM Website by Year**

| **Year 1** | **Users** | **Year 2** | **Users** | **Year 3** | **Users** | **Year 4** | **Users** | **Year 5** | **Users** | **Year 6** | **Users** |
| --- | --- | --- | --- | --- | --- | --- | --- | --- | --- | --- | --- |
| 1. UK | 3673 | 1. UK | 10956 | 1. US | 23408 | 1. US | 42107 | 1. US | 61012 | 1. US | 79928 |
| 1. US | 2566 | 1. US | 9002 | 1. UK | 22004 | 1. UK | 36257 | 1. UK | 52375 | 1. UK | 65417 |
| 1. India | 796 | 1. India | 1971 | 1. Australia | 4770 | 1. Australia | 8472 | 1. Australia | 12037 | 1. Australia | 15487 |
| 1. New Zealand | 551 | 1. Australia | 1926 | 1. India | 4304 | 1. India | 7272 | 1. India | 10239 | 1. India | 13895 |
| 1. Australia | 437 | 1. Canada | 1212 | 1. Canada | 3450 | 1. Canada | 6084 | 1. Canada | 8612 | 1. Canada | 10796 |
| 1. Brazil | 259 | 1. New Zealand | 1089 | 1. New Zealand | 1630 | 1. Malaysia | 2650 | 1. Malaysia | 4209 | 1. Malaysia | 5788 |
| 1. Canada | 257 | 1. Malaysia | 573 | 1. Malaysia | 1420 | 1. New Zealand | 2379 | 1. Ireland | 3683 | 1. Ireland | 4825 |
| 1. China | 174 | 1. Ireland | 546 | 1. Ireland | 1339 | 1. Ireland | 2350 | 1. New Zealand | 3142 | 1. New Zealand | 3840 |
| 1. Ireland | 160 | 1. Brazil | 515 | 1. Saudi Arabia | 1131 | 1. Saudi Arabia | 1946 | 1. Saudi Arabia | 2809 | 1. Saudi Arabia | 3534 |
| 1. Germany | 157 | 1. Saudi Arabia | 509 | 1. South Africa | 964 | 1. South Africa | 1660 | 1. Philippines | 2360 | 1. Philippines | 3050 |
| 1. Japan | 147 | 1. Germany | 352 | 1. Philippines | 818 | 1. Philippines | 1606 | 1. South Africa | 2330 | 1. South Africa | 2836 |
| 1. Saudi Arabia | 127 | 1. South Africa | 347 | 1. Brazil | 779 | 1. Pakistan | 1205 | 1. Indonesia | 1770 | 1. Pakistan | 2343 |
| 1. Malaysia | 117 | 1. Philippines | 302 | 1. Pakistan | 641 | 1. Indonesia | 1084 | 1. Pakistan | 1723 | 1. Indonesia | 2187 |
| 1. Italy | 110 | 1. Thailand | 285 | 1. Thailand | 603 | 1. Thailand | 1021 | 1. Singapore | 1589 | 1. Singapore | 2163 |
| 1. South Korea | 107 | 1. South Korea | 284 | 1. Indonesia | 552 | 1. Brazil | 1013 | 1. Thailand | 1449 | 1. Thailand | 1879 |
| 1. South Africa | 97 | 1. Netherlands | 274 | 1. Singapore | 550 | 1. Singapore | 1006 | 1. Egypt | 1285 | 1. Egypt | 1760 |
| 1. Netherlands | 93 | 1. Egypt | 256 | 1. Germany | 539 | 1. Egypt | 889 | 1. Brazil | 1266 | 1. UAE | 1491 |
| 1. France | 74 | 1. UAE | 250 | 1. UAE | 518 | 1. UAE | 848 | 1. UAE | 1173 | 1. Brazil | 1488 |
| 1. UAE | 69 | 1. Japan | 237 | 1. Egypt | 517 | 1. Germany | 793 | 1. Netherlands | 1096 | 1. Netherlands | 1431 |
| 1. Spain | 69 | 1. Italy | 236 | 1. South Korea | 505 | 1. South Korea | 766 | 1. Hong Kong | 1081 | 1. Hong Kong | 1346 |
| 1. Egypt | 65 | 1. Pakistan | 230 | 1. Netherlands | 470 | 1. Netherlands | 735 | 1. South Korea | 1049 | 1. Germany | 1337 |
| 1. Russia | 61 | 1. China | 218 | 1. Spain | 380 | 1. Hong Kong | 710 | 1. Germany | 1031 | 1. South Korea | 1284 |
| 1. Pakistan | 49 | 1. Indonesia | 208 | 1. Italy | 362 | 1. Nigeria | 622 | 1. Nigeria | 942 | 1. Nigeria | 1151 |
| 1. Indonesia | 45 | 1. Singapore | 197 | 1. Hong Kong | 336 | 1. Turkey | 554 | 1. Kenya | 808 | 1. Kenya | 1072 |
| 1. Turkey | 44 | 1. Spain | 186 | 1. Japan | 322 | 1. France | 545 | 1. Spain | 764 | 1. Turkey | 1001 |
| Total Overall: 12,286 |  | Total Overall: 37,342 |  | Total Overall: 82,816 |  | Total Overall: 141,897 | | Total Overall: 204,545 | | Total Overall: 262,476 | |
| Total Countries N= 138 | | Total Countries N= 172 | | Total Countries N=193 | | Total Countries N=204 | | Total Countries N=212 | | Total Countries N=214 | |

*Google Analytic Data from 14^th^ November 2014 (go live date) to 31^st^ July 2020*
