# Supplementary material for: A mixed methods evaluation of the Paediatric Musculoskeletal Matters (PMM) online portfolio
Source: Pediatr Rheumatol Online J. 2021 Jun 9;19:85. doi: 10.1186/s12969-021-00567-5 (PMC8188761; doi:10.1186/s12969-021-00567-5)
Supplement: Supplementary file 3 — Additional file 3. pGALS App (iOS Version) Downloads by Country. Supplementary Table 2 to further illustrate results. [file 12969_2021_567_MOESM3_ESM.docx]

**Additional Table 2: pGALS App (iOS Version) Downloads by Country**

| **Country** | **n** | **Country** | **n** | **Country** | **n** |
| --- | --- | --- | --- | --- | --- |
| China* | 2,372 | Sweden | 61 | Hungary | 10 |
| UK | 1,498 | New Zealand | 56 | France* | 8 |
| US | 706 | Denmark | 29 | Oman | 8 |
| Canada | 454 | Germany | 29 | Taiwan* | 8 |
| Australia | 446 | Italy | 28 | Lithuania | 7 |
| Mexico | 422 | Turkey | 20 | Philippines | 7 |
| Brazil | 330 | Japan | 19 | Chile* | 6 |
| South Africa | 213 | Indonesia | 18 | Egypt | 6 |
| Ireland | 175 | UAE | 18 | Finland | 6 |
| Thailand | 165 | Spain | 17 | Russia | 6 |
| Malaysia | 161 | Croatia | 15 | Trinidad & Tobago | 6 |
| Netherlands | 143 | Belgium | 14 | Malta* | 5 |
| Hong Kong | 117 | Switzerland | 14 | Pakistan | 5 |
| Colombia | 88 | Singapore | 13 | Qatar | 5 |
| Saudi Arabia | 72 | Slovenia | 12 | Slovakia* | 5 |
| India | 69 | Portugal | 11 | Cambodia | 4 |
| Latvia | 68 | Romania | 11 | Greece | 4 |
| Norway | 64 | Argentina | 10 | Czech Republic* | 3 |
| **Total Responses n=8,067 iOS downloads**  **Total Countries n=54**  * unique to iOS | | | | | |

*Analytic Data from 31^st^ July 2020*
