# Supplementary material for: A mixed methods evaluation of the Paediatric Musculoskeletal Matters (PMM) online portfolio
Source: Pediatr Rheumatol Online J. 2021 Jun 9;19:85. doi: 10.1186/s12969-021-00567-5 (PMC8188761; doi:10.1186/s12969-021-00567-5)
Supplement: Supplementary file 4 — Additional file 4. pGALS App (Android Version) Downloads by Country. Supplementary Table 3 to further illustrate results. [file 12969_2021_567_MOESM4_ESM.docx]

**Additional Table 3: pGALS App (Android Version) Downloads by Country**

| **Country** | **n** | **Country** | **n** | **Country** | **n** |
| --- | --- | --- | --- | --- | --- |
| UK | 940 | Norway | 30 | Finland | 10 |
| Mexico | 756 | Singapore | 27 | Libya* | 9 |
| India | 358 | Myanmar* | 26 | Philippines | 9 |
| Brazil | 310 | Sri Lanka* | 24 | Trinidad & Tobago | 9 |
| South Africa | 176 | Sweden | 22 | Ghana* | 8 |
| Australia | 170 | Hungary | 21 | Kenya* | 8 |
| Colombia | 170 | Egypt | 20 | Sudan* | 8 |
| Malaysia | 147 | Belgium | 17 | Cambodia | 6 |
| Canada | 142 | Spain | 17 | Japan | 6 |
| US | 119 | Argentina | 15 | Uruguay* | 6 |
| Latvia | 115 | UAE | 15 | Russia | 5 |
| Hong Kong | 106 | Denmark | 14 | Switzerland | 5 |
| Saudi Arabia | 78 | Germany | 14 | Bulgaria* | 4 |
| Ireland | 72 | Lithuania | 14 | Austria* | 3 |
| Indonesia | 70 | Portugal | 14 | Israel* | 3 |
| Netherlands | 61 | Oman | 13 | Bahamas* | 2 |
| Thailand | 58 | Greece | 12 | Jamaica* | 2 |
| New Zealand | 52 | Poland* | 12 | Jordon* | 2 |
| Croatia | 42 | Romania | 11 | Nigeria* | 2 |
| Pakistan | 35 | Turkey | 11 | Qatar | 2 |
| Slovenia | 31 | Italy | 10 | Brunei* | 1 |
| **Total Responses n= 4,603** android downloads**  **Total Countries n= 63**  * unique to android  ** Country data not available for 126 | | | | | |

*Analytic Data from 31^st^ July 2020*
