# Supplementary material for: A mixed methods evaluation of the Paediatric Musculoskeletal Matters (PMM) online portfolio
Source: Pediatr Rheumatol Online J. 2021 Jun 9;19:85. doi: 10.1186/s12969-021-00567-5 (PMC8188761; doi:10.1186/s12969-021-00567-5)
Supplement: Supplementary file 5 — Additional file 5. Job Title of Registered Users of ELM. Supplementary Table 4 to further illustrate results. [file 12969_2021_567_MOESM5_ESM.docx]

**Additional Table 4: Job Title of Registered Users of ELM**

| **Job title** | **n** |
| --- | --- |
| **Training Doctor** | **36** |
| Medical Student | 12 |
| Paediatric registrar/ General paediatrician trainee | 14 |
| Family medicine doctor trainee | 1 |
| Resident | 3 |
| Senior house officer | 1 |
| Advanced Clinical Practitioner | 1 |
| Clinical Fellow | 1 |
| Doctor. Trainee in Dermatology | 1 |
| Early Stimulation Coordinator | 1 |
| F1 clinical | 1 |
| **Clinician** | **35** |
| General paediatrician | 5 |
| Consultant | 3 |
| Paediatric rheumatologist | 4 |
| Doctor | 12 |
| Family medicine doctor | 8 |
| Orthopaedics | 1 |
| Specialist | 1 |
| GP retainer | 1 |
| **Training Allied Health Professional** | **4** |
| Occupational Therapy Student | 1 |
| Physician Associate Student | 1 |
| Trainee Advanced Nurse Practitioner | 1 |
| Trainee Advanced Practitioner | 1 |
| **Nurse and Allied Health Professional** | **50** |
| Nurse/Nurse Practitioner | 7 |
| Physiotherapist/ Physical therapist | 23 |
| Podiatrist | 16 |
| Occupational Therapist | 1 |
| Orthotist | 1 |
| Paramedic | 2 |
| **Education** | **17** |
| Assistant Professor | 2 |
| Associate Professor | 1 |
| Clinical Lecturer/ lecturer | 3 |
| Professor | 2 |
| Student | 9 |
| **Non Clinical** | **4** |
| Senior Innovation Associate | 1 |
| Senior Learning Technology Advisor | 1 |
| Professional Engagement Officer | 1 |
| Manager | 1 |
| **Total Responses n=146** (4 did not provide this detail) | |

*Analytic Data from 30^th^ May 2019*
