# Supplementary material for: A mixed methods evaluation of the Paediatric Musculoskeletal Matters (PMM) online portfolio
Source: Pediatr Rheumatol Online J. 2021 Jun 9;19:85. doi: 10.1186/s12969-021-00567-5 (PMC8188761; doi:10.1186/s12969-021-00567-5)
Supplement: Supplementary file 6 — Additional file 6. ELM Uptake by Country. Supplementary Table 5 to further illustrate results. [file 12969_2021_567_MOESM6_ESM.docx]

**Additional Table 5: ELM Uptake by Country**

| **Country** | **n** | **Country** | **n** |
| --- | --- | --- | --- |
| UK | 91 | Israel | 1 |
| Ireland | 10 | Italy | 1 |
| India | 7 | Kenya | 1 |
| Brazil | 4 | Kuwait | 1 |
| Australia | 3 | Mexico | 1 |
| Malaysia | 3 | Netherlands | 1 |
| Pakistan | 3 | New Zealand | 1 |
| Saudi Arabia | 3 | Nigeria | 1 |
| Canada | 2 | Philippines | 1 |
| US | 2 | Russia | 1 |
| Barbados | 1 | Spain | 1 |
| Bulgaria | 1 | Sri Lanka | 1 |
| Chile | 1 | Sweden | 1 |
| Ethiopia | 1 | Turkey | 1 |
| Germany | 1 | Uruguay | 1 |
| **Total Responses n= 148** (2 did not provide this detail) | | | |

*Analytic Data from 30^th^ May 2019*
