# Supplementary material for: A mixed methods evaluation of the Paediatric Musculoskeletal Matters (PMM) online portfolio
Source: Pediatr Rheumatol Online J. 2021 Jun 9;19:85. doi: 10.1186/s12969-021-00567-5 (PMC8188761; doi:10.1186/s12969-021-00567-5)
Supplement: Supplementary file 7 — Additional file 7. Survey Respondent Job Title or Course of Study. Supplementary Table 6 to further illustrate results. [file 12969_2021_567_MOESM7_ESM.docx]

**Additional Table 6: Survey Respondent Job Title or Course of Study**

|  | **PMM Website** | | | **pGALS App** | | | **ELM** | | |
| --- | --- | --- | --- | --- | --- | --- | --- | --- | --- |
| **Job title or course of study as defined by the respondent** | **User**  **n (%)** | **Non user**  **n (%)** | **Overall**  **n (%)** | **User**  **n (%)** | **Non user**  **n (%)** | **Overall**  **n (%)** | **User**  **n (%)** | **Non user**  **n (%)** | **Overall**  **n (%)** |
| **Training Doctor** |  |  |  |  |  |  |  |  |  |
| Medical student | 5 (4.85%) | 2 (3.28%) | 7 (4.27%) | 4 (8.33%) | 2 (2.41%) | 6 (4.58%) | 4 (8%) | 2 (2.74%) | 6 (4.88%) |
| General paediatrics trainee | 3 (2.91%) | 0 | 3 (1.83%) | 1 (2.08%) | 2 (2.41%) | 3 (2.29%) | 0 | 1 (1.37%) | 1 (0.81%) |
| Paediatric rheumatology trainee | 4 (3.88%) | 1 (1.64%) | 5 (3.05%) | 4 (8.33%) | 1 (1.20%) | 5 (3.82%) | 1 (2%) | 4 (5.48%) | 5 (4.07%) |
| Family medicine doctor trainee | 1 (0.97%) | 0 | 1 (0.61%) | 1 (2.08%) | 0 | 1 (0.76%) | 1 (2%) | 0 | 1 (0.81%) |
| **Clinician** |  |  |  |  |  |  |  |  |  |
| General paediatrician | 13 (12.62%) | 5 (8.20%) | 18 (10.98%) | 7 (14.58%) | 7 (8.43%) | 14 (10.69%) | 3 (6%) | 10 (13.70%) | 13 (8.94%) |
| Paediatric rheumatologist | 16 (15.53%) | 3 (4.92%) | 19 (11.59%) | 11 (22.92%) | 4 (4.82%) | 15 (11.45%) | 3 (6%) | 12 (16.44%) | 15 (12.20%) |
| Family medicine doctor | 4 (3.88%) | 0 | 4 (2.44%) | 1 (2.08%) | 3 (3.61%) | 4 (3.05%) | 3 (6%) | 1 (1.37%) | 4 (3.25%) |
| Orthopaedic surgeon | 1 (0.97%) | 0 | 1 (0.61%) | 0 | 1 (1.20%) | 1 (0.76%) | 0 | 1 (1.37%) | 1 (0.81%) |
| Clinical lecturer/ Research fellow/ Medical laboratory | 3 (2.91%) | 0 | 3 (1.83%) | 2 (4.17%) | 0 | 2 (1.53%) | 1 (2%) | 1 (1.37%) | 2 (1.63%) |
| **Nurse and Allied Health Professional** |  |  |  |  |  |  |  |  |  |
| Allied health professional  *(includes: physiotherapists, podiatrists, occupational therapists, extended scope practitioner & additional needs practitioner)* | 49 (47.57%) | 48 (78.69%) | 97 (59.15%) | 17 (35.42%) | 59 (71.08%) | 76 (58.02%) | 33 (66%) | 38 (52.05%) | 71 (57.72%) |
| Nurse/nurse practitioner | 4 (3.88%) | 2 (3.28%) | 6 (3.66%) | 0 | 4 (4.82%) | 4 (3.05%) | 1 (2%) | 3 (4.11%) | 4 (3.25%) |
| **Total *** | **n=103** | **n=61** | **n=164** | **n= 48** | **n=83** | **n=131** | **n=50** | **n=73** | **n=123** |

*164 respondents completed the survey. Of these, 120 provided feedback on all three resources within the PMM portfolio, 10 on PMM website and pGALS app, 3 on PMM website and ELM, and 31 on PMM website alone.
