# Supplementary material for: A mixed methods evaluation of the Paediatric Musculoskeletal Matters (PMM) online portfolio
Source: Pediatr Rheumatol Online J. 2021 Jun 9;19:85. doi: 10.1186/s12969-021-00567-5 (PMC8188761; doi:10.1186/s12969-021-00567-5)
Supplement: Supplementary file 8 — Additional file 8. Countries Survey Respondent Reside In. Supplementary Table 7 to further illustrate results. [file 12969_2021_567_MOESM8_ESM.docx]

**Additional Table 7: Countries Survey Respondent Reside In**

|  | **PMM Website** | | | **pGALS App** | | | **ELM** | | |
| --- | --- | --- | --- | --- | --- | --- | --- | --- | --- |
| **Country** | **User**  **n (%)** | **Non user**  **n (%)** | **Overall**  **n (%)** | **User**  **n (%)** | **Non user**  **n (%)** | **Overall**  **n (%)** | **User**  **n (%)** | **Non user**  **n (%)** | **Overall**  **n (%)** |
| Argentina | 2 (2.04%) | 0 | 2 (1.27%) | 2 (4.55%) | 0 | 2 (1.61%) | 0 | 2 (2.78%) | 2 (1.72%) |
| Brazil | 4 (4.08%) | 1 (1.69%) | 5 (3.18%) | 4 (9.09%) | 1 (1.25%) | 5 (4.03%) | 1 (2.27%) | 4 (5.56%) | 5 (4.31%) |
| Canada | 0 | 1 (1.69%) | 1 (0. 64%) | 1 (2.27%) | 0 | 1 (0.81%) | 1 (2.27%) | 0 | 1 (0.86%) |
| Colombia | 1 (1.02%) | 0 | 1 (0. 64%) | 1 (2.27%) | 0 | 1 (0.81%) | 1 (2.27%) | 0 | 1 (0.86%) |
| Egypt | 1 (1.02%) | 1 (1.69%) | 2 (1.27%) | 0 | 1 (1.25%) | 1 (0.81%) | 0 | 1 (1.39%) | 1 (0.86%) |
| Finland | 1 (1.02%) | 0 | 1 (0. 64%) | 1 (2.27%) | 0 | 1 (0.81%) | 0 | 1 (1.39%) | 1 (0.86%) |
| Greece | 1 (1.02%) | 0 | 1 (0. 64%) | 0 | 1 (1.25%) | 1 (0.81%) | 0 | 1 (1.39%) | 1 (0.86%) |
| Hungary | 2 (2.04%) | 0 | 2 (1.27%) | 0 | 1 (1.25%) | 1 (0.81%) | 0 | 1 (1.39%) | 1 (0.86%) |
| India | 38 (38.78%) | 31 (52.54%) | 69 (43.95%) | 13 (29.55%) | 39 (48.75%) | 52 (41.94%) | 21 (47.73%) | 30 (41.67%) | 51 (43.97%) |
| Indonesia | 3 (3.06%) | 0 | 3 (1.91%) | 2 (4.55%) | 0 | 2 (1.61%) | 0 | 2 (2.78%) | 2 (1.72%) |
| Italy | 1 (1.02%) | 0 | 1 (0. 64%) | 1 (2.27%) | 0 | 1 (0.81%) | 0 | 1 (1.39%) | 1 (0.86%) |
| Malaysia | 7 (7.14%) | 1 (1.69%) | 8 (5.10%) | 5 (11.36%) | 1 (1.25%) | 6 (4.84%) | 5 (11.36%) | 1 (1.39%) | 6 (5.17%) |
| Mexico | 1 (1.02%) | 0 | 1 (0. 64%) | 0 | 1 (1.25%) | 1 (0.81%) | 1 (2.27%) | 0 | 1 (0.86%) |
| Nigeria | 0 | 2 (3.39%) | 2 (1.27%) | 0 | 2 (2.50%) | 2 (1.61%) | 0 | 2 (2.78%) | 2 (1.72%) |
| Oman | 1 (1.02%) | 0 | 1 (0. 64%) | 0 | 0 | 0 | 0 | 0 | 0 |
| Pakistan | 8 (8.16%) | 10 (16.95%) | 18 (11.46%) | 2 (4.55%) | 14 (17.50%) | 17 (13.71%) | 6 (13.64%) | 6 (8.33%) | 12 (10.34%) |
| Saudi Arabia | 0 | 1 (1.69%) | 1 (0. 64%) | 0 | 1 (1.25%) | 1 (0.81%) | 0 | 1 (1.39%) | 1 (0.86%) |
| Singapore | 1 (1.02%) | 0 | 1 (0. 64%) | 0 | 1 (1.25%) | 1 (0.81%) | 0 | 1 (1.39%) | 1 (0.86%) |
| South Africa | 1 (1.02%) | 0 | 1 (0. 64%) | 1 (2.27%) | 0 | 1 (0.81%) | 0 | 1 (1.39%) | 1 (0.86%) |
| Spain | 0 | 1 (1.69%) | 1 (0. 64%) | 0 | 1 (1.25%) | 1 (0.81%) | 0 | 1 (1.39%) | 1 (0.86%) |
| Syria | 1 (1.02%) | 0 | 1 (0. 64%) | 1 (2.27%) | 0 | 1 (0.81%) | 0 | 1 (1.39%) | 1 (0.86%) |
| Thailand | 2 (2.04%) | 0 | 2 (1.27%) | 2 (4.55%) | 0 | 2 (1.61%) | 0 | 2 (2.78%) | 2 (1.72%) |
| UAE | 0 | 3 (5.08%) | 3 (1.91%) | 0 | 3 (3.75%) | 3 (2.42%) | 1 (2.27%) | 1 (1.39%) | 2 (1.72%) |
| UK | 21 (21.43%) | 7 (11.86%) | 28 (17.83%) | 7 (15.91%) | 13 (16.25%) | 21 (16.94%) | 7 (15.91%) | 11 (15.28%) | 18 (15.52%) |
| US | 1 (1.02%) | 0 | 1 (0.64%) | 1 (2.27%) | 0 | 1 (0.81%) | 0 | 1 (1.39%) | 1 (0.86%) |
| Total | n= 98  (5 did not answer this question) | n=59  (2 did not answer this question) | n=157  (7 did not answer this question) | n= 44  (4 did not answer this question) | n=80  (3 did not answer this question) | n=124  (7 did not answer this question) | n=44  (6 did not answer this question) | n=72  (1 did not answer this question) | n=116  (7 did not answer this question) |
